# Deep Brain Stimulation Impact on Social and Occupational Functioning in Parkinson's Disease with Early Motor Complications

**DOI:** 10.1002/mdc3.13015

**Published:** 2020-08-03

**Authors:** Valerie Stoker, Paul Krack, Lisa Tonder, Gillian Barnett, Isabelle Durand‐Zaleski, Alfons Schnitzler, Jean‐Luc Houeto, Lars Timmermann, Joern Rau, Carmen Schade‐Brittinger, Marie Vidailhet, Günther Deuschl, EARLYSTIM Study Group

**Affiliations:** ^1^ Medtronic Minneapolis Minnesota USA; ^2^ Department of Neurology University Hospital Bern and University of Bern Bern Switzerland; ^3^ University of Paris Paris France; ^4^ Institute of Clinical Neuroscience and Medical Psychology, and Department of Neurology Heinrich‐Heine University Duesseldorf Duesseldorf Germany; ^5^ Department of Neurology, Clinical Investigational Centers‐Institut National de la Sante et de la Recherche Medicale 1402, Centre Hospitalier Universitaire of Poitiers University of Poitiers Poitiers France; ^6^ Universitätsklinikum Giessen und Marburg Marburg Germany; ^7^ The Coordinating Center for Clinical Trials Philipps University Marburg Germany; ^8^ Sorbonne Université, Insitut du Cerveau et de la Moelle Epinere UMR1127, Insitut National de la Sante et de la Recherche Medicale &1127, Centre National de la Recherche Scientifique 7225, Department of Neurology Salpêtriere University Hospital Assistance Publique – Hôpitaux de Paris Paris France; ^9^ Department of Neurology, Universitätsklinikum Schleswig‐Holstein Kiel Campus Christian‐Albrechts‐University Kiel Germany

**Keywords:** deep brain stimulation, Parkinson's disease, productivity, occupational, psychosocial.

## Abstract

**Background:**

Deep brain stimulation (DBS) improves motor symptoms and quality of life in patients with Parkinson's disease (PD) and early motor complications, suggesting that DBS could be prescribed to the working‐age PD population.

**Objectives:**

To investigate the effect of DBS compared with best medical therapy (BMT) on social, psychosocial, and occupational functioning in patients with PD ≤60 years of age with early motor complications, its correlates, and possible underlying rationale.

**Methods:**

Methods included analyses of the Social and Occupational Functioning Assessment Scale, Scales for Outcomes for Parkinson's–Psychosocial, Professional Fitness, Starkstein Apathy Scale, and Schwab and England Activities of Daily Living Scale from the EARLYSTIM study.

**Results:**

Compared with BMT, DBS resulted in significantly greater improvements from baseline through 24 months in social,occupational, and psychosocial functioning. Yet, work status in the 2 groups did not differ at baseline and 24 months. Physicians reported a significantly higher percentage of patients in the BMT group unable to work at 24 months relative to baseline compared with the DBS group. Apathy was significantly worse in patients for whom physicians overrated ability to work when compared with patients’ own ratings than in the group of patients who physicians' ability to work ratings were comparable to, or worse than, patients' self‐ratings of ability to work.

**Conclusions:**

For patients aged ≤60 years with PD and early motor complications, DBS provided significant improvements in social, occupational, and psychosocial function, but not in the actual work engagement compared with BMT at 2 years. Apathy may impact ability to work.

Deep brain stimulation (DBS) of the subthalamic nucleus is an established treatment for patients with Parkinson's disease (PD) with fluctuations and dyskinesia refractory to medical therapy.^1^, ^2^, ^3^, ^4^, ^5^, ^6^ Eligible patients typically undergo DBS implantation 11 to 13 years (mean values) after medical therapy has been prescribed,^1^, ^2^, ^3^, ^7^ when severe motor complications have markedly reduced quality of life and patients have reached retirement age. However, with a disease prevalence of 107 per 100,000 persons aged 50 to 59 years,^8^ and 32% of patients with PD with disease onset before the age of 59 years,^9^ PD is not uncommon among working‐age individuals. Indeed, more than half (56%) of nonretired persons with PD onset before the age of 50 living in the United Kingdom were unemployed as a result of disability,^10^ and persons with PD retire 4 to 7 years earlier than the general population.^11^

PD reduces productivity while at work (presenteeism) and increases absenteeism from work and work activity impairment compared with controls.^12^ In addition, diminished capacity to work contributes to the financial burden of PD with up to 49% of the total cost arising from the indirect cost of reduced work capacity and early retirement in the United States and Europe.^13^, ^14^

When evaluating new health care technologies, reimbursement agencies have advocated the adoption of a broad societal perspective in economic analyses by considering the cost of productivity loss and early retirement in complementary analyses by agencies in France and Germany and in primary analyses in Scandinavia and the Netherlands.^15^, ^16^ To date, the societal impact of DBS has only been described in the results of a single‐center retrospective study in Hungary^17^ showing that for employed patients with PD, DBS may help preserve working capability.

The EARLYSTIM study was designed to evaluate the impact of DBS compared with best medical therapy (BMT) on quality of life, the primary endpoint, and multiple secondary endpoints for patients with PD with recent onset motor complications (≤3 years), preserved psychosocial competence, and a younger age (≤60 years) to reflect a working‐age population.^18^ To address knowledge gaps, the objectives of this secondary analysis were to investigate the effect of DBS compared with BMT on social, psychosocial, and occupational functioning in working‐age patients with PD with early motor complications, its correlates, and possible underlying rationale.

## Methods

The design and main results of the EARLYSTIM study (NCT00354133) are described elsewhere.^18^, ^19^ In brief, 251 patients in France and Germany with PD and early levodopa‐induced motor complications with a mean age of 52 years who were, on average, 7.5 years post‐PD onset, were randomized to DBS plus BMT (the DBS group) or BMT alone (the BMT group). Patients were assessed at baseline and 5, 12, and finally, 24 months after randomization; albeit some endpoints were only at assessed at baseline and 24 months.

For this analysis, endpoints assessed in the EARLYSTIM study, including the Social and Occupational Functioning Assessment Scale (SOFAS), the Scales for Outcomes for Parkinson's–Psychosocial (SCOPA‐PS), and the Professional Fitness questionnaire (to determine ability to work and work status), were analyzed.

Correlations between social and occupational functioning and activities of daily living (using the modified Schwab and England Activities of Daily Living Scale [Schwab and England]) and patient apathy (using the Starkstein Apathy Scale) by categories of physician versus patient ratings of ability to work were also analyzed. Additional results of SCOPA‐PS, the Starkstein Apathy Scale, and Schwab and England are briefly reported elsewhere.^19^ Instruments with the exception of the Professional Fitness questionnaire have acceptable psychometric properties in terms of reliability and validity.

SOFAS is a quantitative scale which assesses psychosocial competence in terms of social and occupational functioning.^20^ It is scored as a percentage (in increments of 10 percentage points) on a continuum ranging from grossly impaired functioning (persistent hygiene problems) to excellent functioning (superior functioning in a wide range of activities). The SCOPA‐PS was administered at study visits to assess psychosocial constraints brought about by PD in the previous month.^21^ It is scored on a scale of 0 to 33; a higher score indicates worse functioning. The Professional Fitness questionnaire requests patients and physicians select from the following 4 categories of ability to work: work full‐time without limitations, work with limitations but full‐time, work with significant limitations in a part‐time position, and not able to work. Actual work status at all time points was also collected with the Professional Fitness questionnaire where work was defined as engagements with salary and work‐like engagements without salary (eg, taking care of grandchildren on a regular basis replacing a profession). Patients were also instructed to classify their work status into the following categories based on the German social security system: full‐time work, half‐time work, less than half‐time work, or not working.^22^ The patient‐reported Starkstein Apathy Scale comprises 14 questions, each is scored from 0 to 3, with a maximum of 42 points; a higher score indicates more severe apathy.^23^ The Schwab and England is an assessment of activities of daily living relative to complete independence based on an interview referring to the week before the study visit. Responses reflect the “best” state and “worst’ state and scores range from 0% (complete dependence) to 100% (complete independence).^24^

### Statistical Methods

Mean change in SOFAS and SCOPA‐PS scores at 24 months were compared with baseline within each treatment group and between groups using mixed model statistical analysis with a normality assumption, the baseline value for baseline adjustment, main effects for group, time, a group‐by‐time interaction, center as random effect, and a generalized covariance matrix (to account for serial dependency among observations).

Responses to the Professional Fitness questions were converted to a binary outcome of “unable to work” versus “able to work” for the between‐group comparison at 24 months, and logistic regression analysis was performed using binomial assumptions, a logit link, and main effects for group, time, a group‐by‐time interaction, and an exchangeable covariance structure. The difference between the physician and patient ratings of ability to work was converted into a categorical variable with 3 levels: physician rating of ability to work is better than the patient rating, physician and patient ratings of ability to work are the same, and physician rating of ability to work is worse than the patient rating. A proportional odds model with a cumulative logit link was used for the analysis with factors for group, visit, and a group‐by‐visit interaction. The physician versus patient ratings for ability to work were evaluated over time with an added covariate of the Starkstein Apathy Scale score at 24 months to investigate the impact of apathy on physicians' ratings of professional fitness relative to patients' self‐assessment. The correlation between Schwab and England and SOFAS scores at 24 months was analyzed using the Pearson product‐moment correlation coefficient. The proportion of patients employed at 24 months and those working at baseline and 24 months for the DBS versus BMT groups were compared using a chi‐square test.

Analysis of the social and occupational endpoints (SOFAS and SCOPA‐PS) included the intention‐to‐treat cohort, whereas the analysis of Professional Fitness and the correlation between SOFAS and Schwab and England included the completers' data set.

Generalized least squares estimations with standard errors, and 95% confidence intervals and *P* values were calculated. Adjustments for multiple comparisons were not implemented. Statistical significance was identified as a *P* value ≤ 0.05.

## Results

Baseline demographic and clinical characteristics of the intention‐to‐treat cohort are described elsewhere.^19^ In summary, 124 patients were assigned to DBS (97% received DBS therapy and completed the study), and 127 patients were assigned to BMT (98% received BMT and 97% completed the study). SOFAS was used to determine social and occupational functioning as one inclusion criterion at study enrollment, with impairment of 51% to 80% (ie, moderate difficulty to slight impairment in social functioning) required. Baseline demographic and clinical characteristics did not differ significantly between the treatment groups, and baseline employment rates (part‐time or full‐time) in the DBS (62.6%) and BMT groups (61.4%) were comparable.

**FIG. 1:**
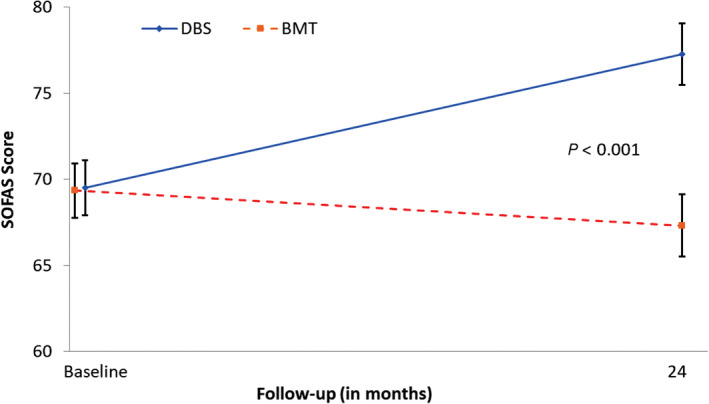
Change in SOFAS Scale. Higher values indicate an improvement as compared with baseline. Data are means ± standard error estimated by mixed model regression. BMT, best medical therapy; DBS, deep brain stimulation; SOFAS, Social and Occupational Functioning Assessment Scale.

Social and occupational functioning (SOFAS score) improved by 11% from baseline to 24 months (*P* < 0.05) in the DBS group, a mean category change from “some difficulty” to “slight impairment.” In the BMT group, it decreased by 3% (*P* > 0.05) through 24 months; however, the BMT group had maintained its baseline mean category status of “some difficulty,” which refers to the categorical assessment based on a score of 61% to 70%, whereas slight impairment is 71% to 80%, and superior functioning 91% to 100%. Compared with BMT, DBS resulted in a greater improvement from baseline through 24 months in social and occupational functioning (mean ± standard error [SE] difference, 9.8 ± 1.9 points; *P* < 0.001) (Fig. 1 and Table 1). There were positive moderate correlations between the best and worst Schwab and England and SOFAS scores at 24 months for both treatment groups (*r*, range, 0.47–0.64; *P* < 0.0001).

**TABLE 1:** Change from baseline to 24 months—SOFAS and SCOPA‐PS

	Baseline				Within‐Treatment Change from Baseline to 24 Months		Between‐Treatment Change from Baseline to 24 Months	
	DBS		BMT		DBS	BMT	Differences	
Endpoint	n	Mean ± SE	N	Mean ± SE	Mean ± SE (% Change)	Mean ± SE (% Change)	Mean ± SE; 95% CI	Significance
SOFAS^a^	124	69.5 ± 1.6	127	69.3 ± 1.6	7.8 ± 1.3 (11)^c^	−2.02 ± 1.3 (−3)	9.8 ± 1.9; 6.3–13.5	*P* < 0.001
SCOPA‐PS^b^	124	9.1 ± 0.5	127	9.0 ± 0.5	−2.5 ± .0.5 (−28)^c^	−0.4 ± 0.5 (−3)	−2.1 ± 0.7; −3.9 to −0.4	*P* = 0.023

^a^ Positive change indicates improvement.

^b^ Negative change indicates improvement.

^c^ Within‐group change, *P* ≤ 0.05.

Abbreviations: SOFAS, Social and Occupational Functioning Assessment Scale; SCOPA‐PS, Scales for Outcomes for Parkinson's–Psychosocial; DBS, deep brain stimulation; BMT, best medical therapy; SE, standard error; CI, confidence interval.

**FIG. 2:**
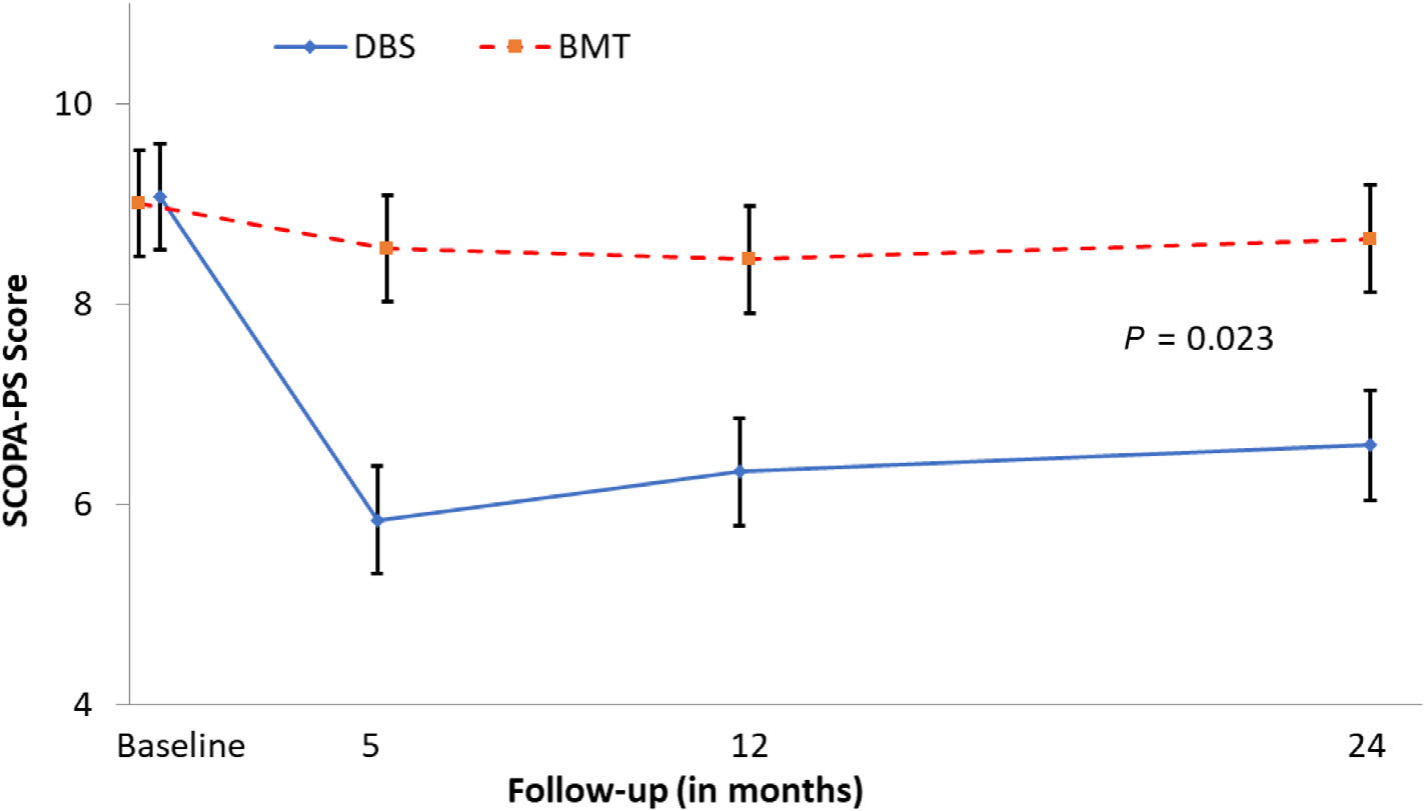
Change in SCOPA‐PS score. Lower values indicate an improvement as compared with baseline. Data are means ± standard error estimated by mixed model regression. BMT, best medical therapy; DBS, deep brain stimulation; SCOPA‐PS, Scales for Outcomes for Parkinson's–Psychosocial.

Psychosocial functioning, measured using the SCOPA‐PS, improved by 28% and 3% from baseline to 24 months in the DBS group (mean ± SE, −2.5 ± 0.5; *P* < 0.05) and the BMT group (mean ± SE, −0.5 ± 0.5; *P* > 0.05), respectively. Compared with BMT, DBS resulted in a greater improvement from baseline through 24 months in psychosocial functioning (mean difference ± SE, −2.1 ± 0.7; *P* = 0.023) (Fig. 2 and Table 1).

**FIG. 3:**
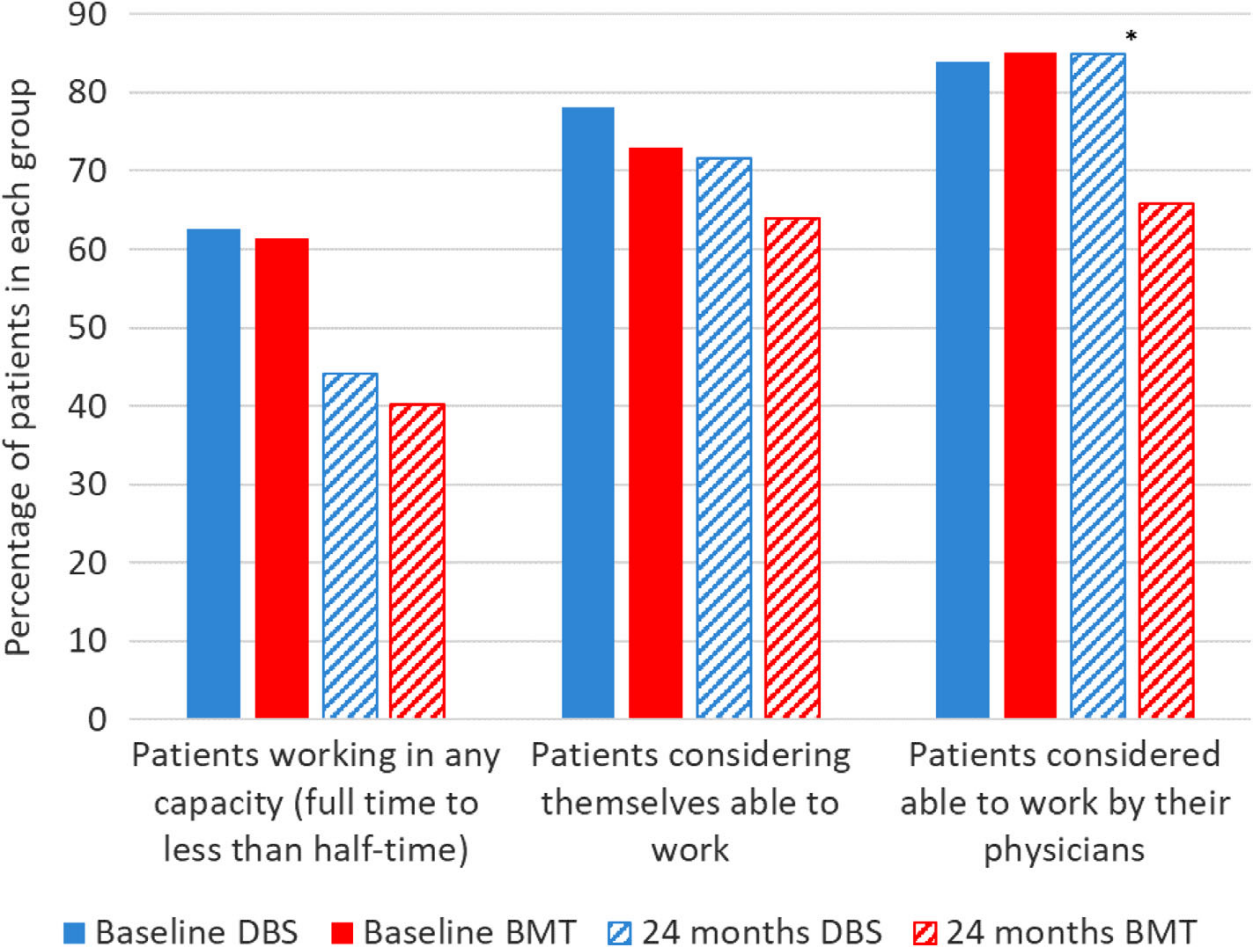
Change in actual work status and ability to work. BMT, best medical therapy; DBS, deep brain stimulation. *Change between groups from baseline to 24 months *P* ≤ 0.05.

**FIG. 4:**
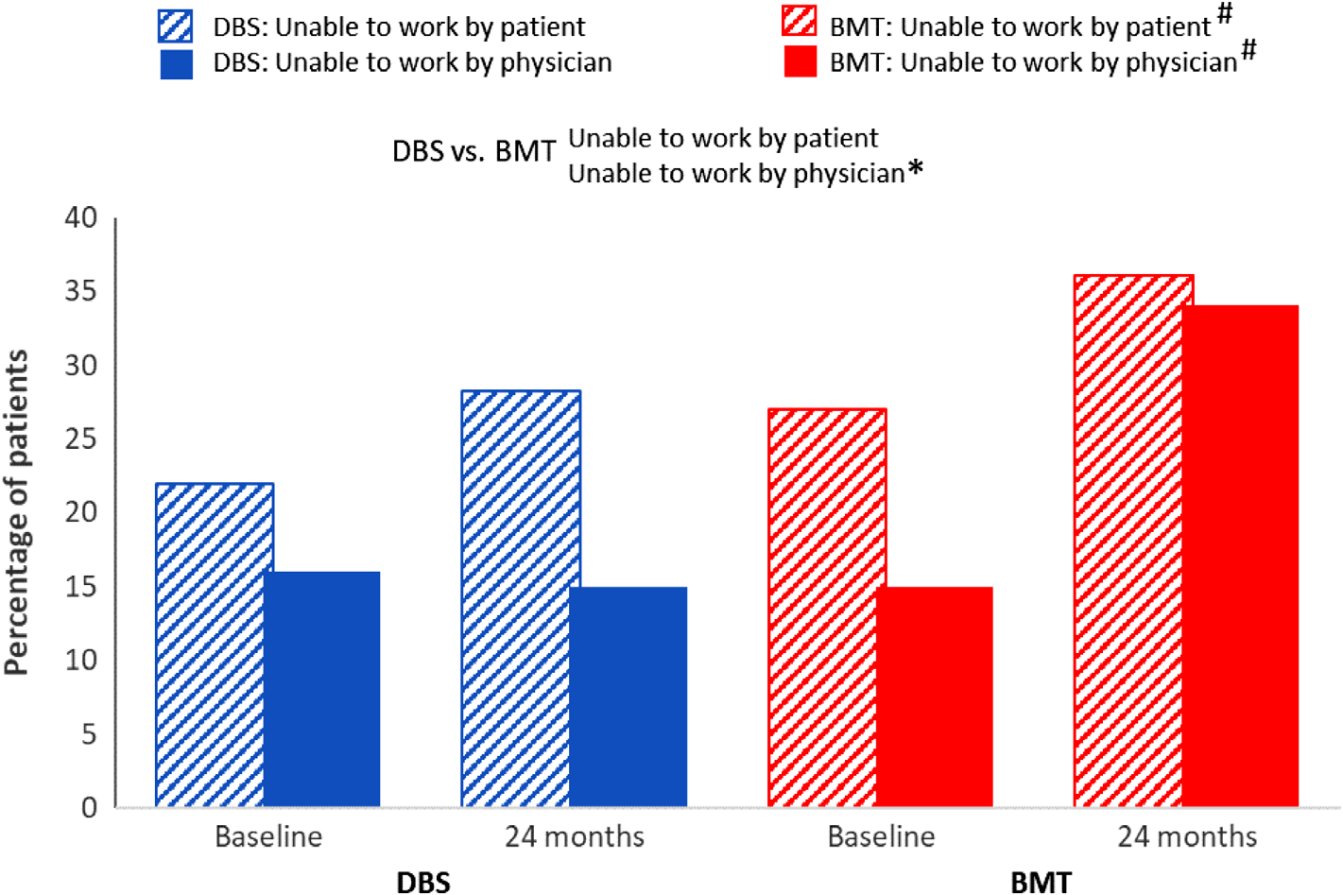
Change in professional fitness ratings—“unable to work.” BMT, best medical therapy; DBS, deep brain stimulation. ^#^ Within‐group change, *P* ≤ 0.05; *between‐group change, *P* ≤ 0.05.

At 24 months, work status (unemployed vs. full‐time employed) was not significantly different between the DBS (44.2%) and BMT (40.2%) groups. Physicians consistently rated patients as more able to work compared with how patients rated themselves (Fig. 3). When physicians' subjective assessment of patients' ability to work in response to the Professional Fitness questionnaire was converted to a binary outcome (“unable to work” vs. “able to work”), there was an increase in the percentage of patients rated as unable to work over 24 months in the BMT group (*P* < 0.001) compared with no change in the DBS group (*P* = 0.789), with a difference between treatment groups at 24 months (*P* = 0.003) in favor of DBS (Fig. 4). Patient self‐assessment of being “unable to work” showed a worsening over 24 months in the BMT group (27% vs. 36%; *P* = .023) and no change in the DBS group (22% vs. 28%; *P* = 0.154). There was no difference in patient self‐assessment between treatment groups at 24 months (*P* = 0.586).

**FIG. 5:**
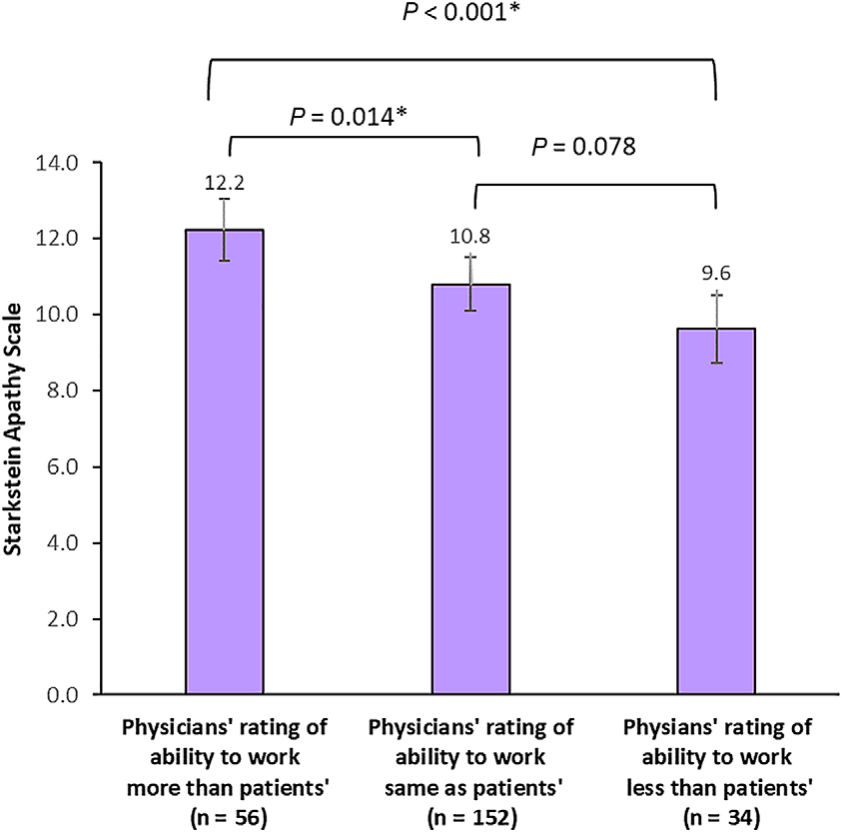
Starkstein Apathy Scale scores by physician versus patient responses to the ability to work at 24 months. Higher score indicates more severe apathy. Data are means ± standard error estimated by mixed model regression. *Difference between categories *P* ≤ 0.05.

Differences in ability to work ratings between physicians and patients at 24 months were further explored in analysis of patients' apathy measured with the Starkstein Apathy Scale. Results showed significantly worse patient‐reported apathy in the group comprising patients whose physician ratings of ability to work were more than their own ratings compared with the patient group in which physicians provided the same ability to work ratings as the patients (Fig. 5). In addition, significantly worse patient‐reported apathy was also recorded in the group in which physicians rated patients more able to work than patients rated themselves compared with the group with physicians who rated patients less able to work than patients rated themselves.

## Discussion

To the best of our knowledge, this is the first publication of social and occupational outcomes of DBS compared with BMT for patients with PD ≤60 years of age with early motor complications based on data from a randomized controlled trial (EARLYSTIM), showing that DBS significantly improved social, occupational, and psychosocial functioning from baseline through 2 years compared with BMT. The improvement in social and occupational functioning with DBS was also clinically meaningful with patients moving from being categorized as having “some difficulty” to a “slight impairment.” The actual work status did not differ between the BMT and DBS groups at baseline and after 24 months. Physicians reported that BMT group patients' ability to work decreased over 2 years, whereas patients in the DBS group maintained their baseline professional fitness, with a statistically significant between‐group difference at 24 months. Another key finding was that physicians viewed patients as being more able to work than patients viewed themselves.

This controlled study also offers the possibility to compare a large group of patients randomized to DBS or BMT for psychosocial outcomes and employment status. First, we found that of the patients who were working at baseline in the DBS group, 69% were successfully retained in the workforce compared with 54% in the BMT group at 24 months (*P* value of 0.052, considering that this group of patients who were working at baseline was 60.2% of the overall cohort, this analysis was not powered to detect a meaningful treatment difference). In EARLYSTIM, with a mean age of 52 years and a 63% employment rate at baseline, despite multiple clinical benefits with DBS 2 years postimplantation,^19^ the employment rate had decreased to 44%. There may be other economic or societal factors influencing actual work status, as even in the general population, employment rates are decreasing with increasing age. The employment rate in Germany and France among the general population aged 25 to 54 years of age in 2018 was 88%, whereas among those aged 55 to 64 years, 75% and 56% were employed, respectively.^19^

In contrast, the ratings by both patients and physicians for ability to work were higher than the actual employment status, with the physicians' ratings being higher than the patients' ratings. The difference between actual employment status and the patients' ratings may be attributed to a lack of opportunity, as it is difficult for persons aged >50 years to find suitable employment in France and Germany, where EARLYSTIM was conducted. The difference between the physicians' and the patients' assessments may indeed reflect factors outside the mere physical and mental abilities of the patients. This has been assessed with different questionnaires exploring psychological and social variables.

Results from our analyses of SOFAS and the Schwab and England, both measures of disability, support greater independence for activities of daily living with DBS treatment.^19^ As expected, social and occupational functioning and ability to perform every day activities were positively correlated; as one improves, so does the other.

Social anxiety and social phobia, common among persons with PD,^25^, ^26^ contribute to reduced social participation^27^ and are strongly related to the impact of reduced mobility on quality of life.^28^ The improvement in psychosocial functioning (SCOPA‐PS) in the DBS group compared with the BMT group is consistent with the findings of social and occupational functioning (SOFAS). Another psychosocial impact of PD is the stigma subscore of the Parkinson's Disease Questionnaire‐39 that improved most with DBS, as described in the EARLYSTIM study main publication.^19^ A possible consequence of stigma is shame, a self‐perception of inadequacy and violation of the norm.^29^ Shame is a relevant psychosocial impact of PD that likely also contributes to a patient's ability to work.

Analysis of data in the national patient registry in Denmark showed that 8 years before PD was diagnosed (typically based on motor symptoms) there were significantly fewer patients employed compared with non‐PD controls.^30^, ^31^ This may reflect the impact of premotor symptoms of PD such as anxiety, depression, and fatigue.^31^ In addition, fatigue and slowness are associated with the inability of persons with PD to successfully continue employment.^11^

Apathy and nonmotor outcomes may impact employability, as interpreted by physicians and patients. Apathy is part of a hypodopaminergic postoperative withdrawal syndrome that also includes depression and anxiety.^32^, ^33^ A clinician may be guided by the relative absence of motor symptoms such as tremor or akinesia, whereas the patient's judgment may be more influenced by subjective lack of motivation (apathy) or a subjective feeling of fatigue (apathy) or more generally by the presence of a hypodopaminergic syndrome, which has been shown to occur as part of a dopamine withdrawal syndrome.^32^, ^33^ Importantly, if recognized, this withdrawal syndrome is reversible with appropriate treatment of apathy (eg, by introducing a D2/D3 dopamine agonist).^34^ Another important message from this analysis relates to the rationale for the difference between physician and patient assessment of the patient's ability to work and the relationship with patient‐reported apathy in the current study. One study^35^, ^36^ reported results from a series of 29 patients who had received DBS at an average age of 52 years resulting in an improvement in motor function but not psychosocial adjustment after 18 to 24 months. They interpreted this finding from a psychological perspective as the result of the patient's maladaptation to PD with this therapy. The patient response to the Professional Fitness ability to work questionnaire in EARLYSTIM could be based on underlying apathy interpreted via the patient ‘s own employability rating, but not the physicans’, resulting in the physician being happy but the patient less so. However, although interpretation was mostly psychological, a possible explanation of this discrepancy between physician and patient estimation of employability may be attributed to the hypodopaminergic syndrome, including apathy based on the Starkstein Apathy Scale and the apathy in the Ardouin scale.^37^ The latter reported no substantial increase in hypodopaminergic disorders in patients assigned DBS compared with those BMT from the EARLYSTIM study.

Importantly, discrepancies between patient‐reported and physician‐reported treatment effects and the importance of determining the latter from the patient's perspective versus the physician's subjective interpretation have long been recognized.^38^ Further work on assessing the societal impact of DBS should also consider the recent suggestion to assess broader concepts of “participation” in society beyond the ability to work to fully capture the societal impact of disease and treatments.^39^, ^40^

There are limitations in the interpretation of the analyses as the Professional Fitness questionnaire used has not undergone clinimetric evaluation. No other instruments were available when this trial was designed, and this questionnaire was developed specifically to address the question whether DBS can have an impact on work status, a question raised by an earlier study.^41^ Still, this analysis reinforces the value of patient‐reported outcomes and explains some important differences with physician‐reported outcomes. A further limitation is that a psychological rationale for the differences in the ability to work such as a modification in patients' lifestyle related to changes in coping strategies rather than changes in motivation was not evaluated in this study.

For patients aged ≤60 years presenting with early motor complications of PD, DBS provided significant improvements in social, occupational, and psychosocial functioning from baseline through 2 years compared with BMT. Actual employment status was not different between the 2 groups at 24 months and contrasts with the differences in physician versus patient rating of inability to work which was associated with increased rates of apathy. During postoperative management of DBS in PD, subjective inability to work may be positively influenced by management of dopaminergic medications,^34^ potentially resulting in a “happy doctor with a happy patient.”

## Author Roles

(1) Research Project: A. Conception, B. Organization, C. Execution; (2) Statistical Analysis: A. Design, B. Execution, C. Review and Critique; (3) Manuscript Preparation: A. Writing of the First Draft, B. Review and Critique.

V.S.: 1A, 1B, 1C, 2C, 3A, 3B

P.K.: 1A, 2B, 3B

L.TO.: 2A, 2B, 3B

G.B.: 1B, 2C, 3A, 3B

I.D.Z.: 2C, 3B

A.S.: 1C, 3C

J.‐L.H.: 1A, 2B, 3B

L.Ti.: 1C, 3C

J.R.: 1C, 2B, 2C, 3B

C.S.‐B.: 1B, 1C, 2C, 3B

M.V.: 1C, 2B.

G.D.: 1A, 1C, 2C, 3B

## Disclosures

**Ethical Compliance Statement:** The trial conformed to the Declaration of Helsinki and all patients provided written informed consent before randomization. Permission was approved by the local ethics committees of all centers. The study was registered at ClinicalTrials.gov (NCT00354133).

**Funding Sources and Conflicts of Interest:** The study was funded by the German Federal Ministry of Education and Research (Klinische Studien 01KG0502), French Programme Hospitalier de Recherche Clinique National (P050909) and Medtronic Inc.

**Financial Disclosures for the Previous 12 Months:** V. Stoker is employed by Medtronic, Inc. P. Krack reports grants from Swiss National Science Foundation, Roger De Spoelberch Foundation, Bertarelli Foundation, Michael J. Fox Foundation, Annemarie Opprecht Foundation, and Parkinson Schweiz; research grants from Boston Scientific and Aleva; lecturing fees paid to employing institution from Boston Scientific; and reimbursement of traveling expenses to scientific meeting by Zambon, all outside the submitted work. L. Tonder and G. Barnett are employed by Medtronic Inc. I. Durand‐Zaleski reports consultancy fees paid by Medtronic for this activity. A. Schnitzler has been serving as a consultant for Medtronic, Boston Scientific, St Jude Medical, and Grünenthal and has received lecture fees from Abbvie, Boston Scientific, St Jude Medical, Medtronic, UCB, MEDA Pharma, Teva Pharma, and GlaxoSmithKline. A. Schnitzler is a government employee and receives through hit institution funding for his research from the German Research Council, the German Ministry of Education and Health, and the Gossweiler Foundation. J.‐L. Houeto has received research grants from Agence National de la Recherche, Association France Parkinson and AbbVie and fees for lectures and consultancies from Medtronic, Zambon, AbbVie, and Lundbeck. L. Timmermann has received in the past year received payments as a consultant for Boston Scientific and has received honoraria as a speaker on symposia sponsored by UCB, Desitin, Boston Scientific, and Abbott. The institution of L. Timmermann, not L. Timmermann personally, received funding by the German Research Foundation, the German Ministry of Education and Research, and the Deutsche Parkinson Vereinigung. Neither L. Timmermann nor any member of his family holds stocks, stock options, patents, or financial interests in any of the previously mentioned companies or their competitors. J. Rau and C. Schade‐Brittinger report no disclosures. M. Vidailhet reports no conflicts of interest. G. Deuschl has received lecture fees from Boston Scientific and has been serving as a consultant for Boston Scientific, Cavion, and Functional Neuromodulation. He received royalties from Thieme publishers. He is a government employee and receives through his institution funding for his research from the German Research Council, the German Ministry of Education and Research, and Medtronic.
